# Continuous Monitoring of Interstitial Fluid Glucose Responses to Endurance Exercise with Different Levels of Carbohydrate Intake

**DOI:** 10.3390/nu15224746

**Published:** 2023-11-10

**Authors:** Chiyori Hiromatsu, Naoto Kasahara, Chao-An Lin, Feifei Wang, Kazushige Goto

**Affiliations:** Graduate School of Sports and Health Science, Ritsumeikan University, Kusatsu 525-8577, Japan; sh0041ve@ed.ritsumei.ac.jp (C.H.); sh0194vf@ed.ritsumei.ac.jp (N.K.); gr0520ep@ed.ritsumei.ac.jp (C.-A.L.); hihiw641@gmail.com (F.W.)

**Keywords:** continuous glucose monitoring, glucose, carbohydrate, football

## Abstract

We compared the 24 h changes in interstitial fluid glucose concentration (IGC) following a simulated soccer match between subjects consuming a high-carbohydrate (HCHO; 8 g/kg BW/day) diet and those consuming a moderate-carbohydrate (MCHO; 4 g/kg BW/day) diet. Eight active healthy males participated in two different trials. The subjects were provided with the prescribed diets from days 1 to 3. On day 3, the subjects performed 90 min (2 bouts × 45 min) of exercise simulating a soccer match. The IGC of the upper arm was continuously monitored from days 1 to 4. No significant difference in the IGC was observed between trials during exercise. The total area under the curve (t-AUC) value during exercise did not significantly differ between the HCHO (9719 ± 305 mg/dL·90 min) and MCHO (9991 ± 140 mg/dL·90 min). Serum total ketone body and beta-hydroxybutyrate concentrations were significantly higher in the MCHO than in the HCHO after a second bout of exercise. No significant differences in the IGC were observed between trials at any time point during the night after exercise (0:00–7:00). In addition, t-AUC value during the night did not significantly differ between the HCHO (32,378 ± 873 mg/dL·420 min) and MCHO (31,749 ± 633 mg/dL·420 min). In conclusion, two days of consuming different carbohydrate intake levels did not significantly affect the IGC during a 90 min simulated soccer match. Moreover, the IGC during the night following the exercise did not significantly differ between the two trials despite the different carbohydrate intake levels (8 vs. 4 g/kg BW/day).

## 1. Introduction

Carbohydrate (CHO) is a major source of energy during prolonged exercise in athletes, and it is recommended to increase CHO intake above the normal level during the few days before competing [1]. Sympathetic nervous system activity and hepatic glycogenolysis are stimulated during moderate or high-intensity exercise. Thus, blood glucose concentrations are maintained during exercise unless hepatic glycogen stores become markedly low [2]. Endurance athletes have a strong capacity to maintain their blood glucose concentrations during prolonged exercise compared to untrained subjects [3]. Moreover, 40% of endurance athletes present with glucose concentrations above the reference range for more than 70% of the time, except 2 h after a meal [4]. Therefore, it appears that blood glucose level is maintained, even when CHO intake is less than the recommended amount among athletes.

The main nutritional factors that induce fatigue during a team sports game (e.g., soccer, rugby, basketball) are depletion of muscle glycogen and hypoglycemia [5]. Insufficient CHO intake by soccer players before a match leads to depleted muscle glycogen, resulting in decreased endurance capacity [6] and decreased sprinting speed during the second half [7]. It has also been reported that consuming additional CHO before and during exercise contributes to maintaining a higher level of blood glucose, which prevents a drop in performance and cognitive skills [8,9]. Therefore, 6–8 g/kg BW CHO intake per 24 h during congested fixture schedules is recommended for soccer players [10]. Moreover, additional CHO is recommended before and during a game [11,12], despite the relative rarity of hypoglycemia during a soccer match [13]. Professional soccer players in England consume only about 4 g/kg BW/day on training days during the in-season period, including the day before a match [14]. As exercise-induced physiological stress causes gastrointestinal disturbance and reduces food intake [15], consuming the recommended amount of CHO during a busy match schedule may not be practical for some athletes.

Several studies [7,16,17,18] have reported changes in muscle glycogen before and after a soccer match, whereas continuous changes in blood glucose during exercise and the recovery period (until the next morning) under insufficient CHO intake remain unclear. In the present study, we compared the 24 h changes in IGC following a simulated soccer match between trials that consumed a high-CHO diet and those that had a moderate-CHO diet. We hypothesized that IGC would not be different during and after exercise (including the night) between the trials.

## 2. Methods

### 2.1. Subjects

Eight males (mean ± standard error, age: 23.9 ± 1.1 years, height: 170.1 ± 2.3 cm, body weight: 63.3 ± 2.9 kg, body mass index: 21.9 ± 0.8 kg/m^2^) participated in the present study. All subjects were physically active with recreational resistance exercise or endurance exercise, but none of them were involved in regular resistance or endurance training within 6 months prior to the study. All subjects were informed of the purpose of the study, the experimental procedures, and the possible risks involved in the study, and written informed consent was obtained. The study was approved by the Ethical Committee for Human Experiments at Ritsumeikan University following the Declaration of Helsinki.

### 2.2. Experimental Overview

**Figure 1:**
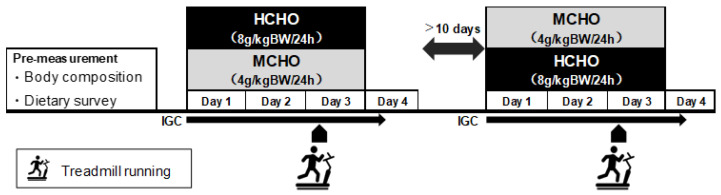
Study protocol. IGC: Interstitial fluid glucose concentration. BW: Body weight. HCHO: High-carbohydrate diet. MCHO: Moderate-carbohydrate diet.

A cross-over design approach was adopted (Figure 1). The main experiment involved two trials consisting of a high-carbohydrate (HCHO; 8 g/kg BW/day CHO) trial and an energy-matched moderate-carbohydrate (MCHO; 4 g/kg BW/day CHO) trial. Subjects initially visited the laboratory for preliminary measurements of height and body weight (BW) using a height and weight meter (WB-510, Tania Inc., Tokyo, Japan). The BW at this time point was utilized for the dietary prescription. A dietary survey was conducted using the food frequency questionnaire (FFQ) before the experiment started to evaluate typical energy and nutrient intake. FFQs are frequently used to assess diet in epidemiological studies and normally include queries on the intake of more than 100 food and beverage items [19]. A FFQ is useful for assessing dietary intake among Japanese [20]. The dietary analysis was conducted using specially designed software (Excel Eiyo-kun version 9.0, Kenpaku-sha, Tokyo, Japan). 

Each trial consisted of 4 consecutive days (days 1–4), separated by a 10-day washout period between trials. Subjects were provided a prescribed diet from days 1 to 3 (until dinner on the exercise day). IGC was continuously monitored from days 1 to 4 (until 9:00 a.m.). Subjects maintained their typical physical activity levels on days 1 and 2. On day 3, they performed 90 min (2 bouts × 45 min) of running exercise on a treadmill to simulate a soccer match. Blood samples were collected three times, before exercise and immediately after completing the first and second bouts of exercise. Acetone levels in exhaled breath were evaluated five times before exercise, after completing the first and second bouts, 30 min after completing the exercise, and the day after exercise (before breakfast).

### 2.3. Dietary Intervention (Days 1–3)

In total, nine prescribed meals were provided from days 1 to 3 and subjects consumed only the prescribed diet at the designated time in each trial. Subjects in the HCHO had the prescribed CHO-rich snacks at 4:00 p.m. on days 1 to 3. In addition, the subjects were allowed to drink water *ad libitum*. The diets were provided as prepackaged food (Nissin Healthcare Food Service Co., Ltd., Tokyo, Japan, and Nosh Co., Ltd., Osaka, Japan) and prepared foods were purchased at a supermarket. Total energy intake was determined by referring to the energy requirement for males (18–29 years old) and physical activity level II in the dietary reference intake for Japanese (ver. 2020). The HCHO diet contained 8 g/kg BW/day CHO, determined based on the recommendation of the Union of European Football Associations (UEFA) expert group [10]. The MCHO diet contained 4 g/kg BW/day CHO, equivalent to the CHO intake of elite football players [14]. Table 1 shows the structure and provision of the diets, ensuring total energy intake and the percentage of macronutrients. The macronutrient contents were calculated using dedicated software (Excel Eiyo-kun version 9.0, Kenpaku-sha) and food labels. On the day of the exercise (day 3), the subjects consumed breakfast containing 2.4 g/kg BW CHO (HCHO) or 1.4 g/kg BW CHO (MCHO) 90 min before starting the exercise, respectively. Subjects had breakfast, lunch, and snacks at the dining space attached to the laboratory and all their meals were consumed. Dinner was taken home and subjects reported how much they consumed. Subjects consumed all of the food provided for both trials.

### 2.4. Exercise Protocol (Day 3)

The subjects conducted a 90 min running exercise simulating a soccer match on a treadmill (Elevation series E95Ta; Life Fitness Corp., Tokyo, Japan; Valiant; Lode, Groningen, the Netherlands). All subjects used the same treadmill for both trials. The exercise protocol was modified from one utilized in previous studies [21,22,23]. The exercise during both trials consisted of two repeated bouts of 45 min (90 min in total) separated by 15 min of rest. Each bout was 9 × 5 min of exercise, including walking (4 km/h) for 90 s, jogging (8 km/h) for 90 s, running (12 km/h) for 75 s, and sprinting (18 km/h) for 45 s. The total running distance covered was 13.95 km in 90 min. The slope of the treadmill was set to 1%. Heart rate (HR) was recorded every 1 min during exercise using a wireless HR monitor (RCX5; Polar Electro, Kempele, Finland). The rating of perceived exertion (RPE) was measured every 5 min while walking at 4 km/h. All subjects completed a 90 min running exercise in both trials.

### 2.5. Measurements

#### 2.5.1. Body Composition

Subjects arrived at the laboratory at 8:00 a.m. after an overnight fast. After rest, body composition was evaluated before eating breakfast on day 1, day 3 (exercise day), and day 4 (the day after the exercise). BW, fat-free mass (FFM), fat mass (FM), and total body water were measured via bio-impedance analysis using a body composition analyzer (InBody 770, In Body Japan Inc., Tokyo, Japan). 

#### 2.5.2. IGC 

The IGC was continuously evaluated by a continuous glucose monitoring (CGM) system (FreeStyle Libre Flash Glucose Monitoring Device, FreeStyle Libre; Abbott Diabetes Care, Alameda, CA, USA; [24,25,26]). The subjects wore the device from day 1 (9:00 a.m.) until day 4 (9:00 a.m.). The sensor was attached to the skin on the back of the upper arm and continuously recorded the average IGC over a 15 min period [27]. Subjects scanned the sensors at intervals of at least 8 h while wearing the device. In addition, the IGC was measured by scanning every 5 min during the 90 min of exercise. A CGM system provides glucose estimates that are comparable with self-monitoring blood concentrations of glucose in normo-glycemic individuals [28]. The Japan Diabetes Society describes that the FreeStyle Libre is available as a medical device for daily self-management of diabetes. 

#### 2.5.3. Levels of Acetone in Exhaled Breath

To evaluate the changes in fat metabolism associated with exercise and the different CHO intake levels, the acetone levels in exhaled breath were determined five times during both trials before starting the exercise, immediately after the first and second bouts, 30 min after completing the exercise, and before breakfast the next day after the exercise (8:00 a.m.). End-tidal air was collected at each measurement time using a special bag. The collected acetone was analyzed by gas chromatography (SGEA-PA-A, Nissha Co., Ltd., Kyoto, Japan). 

#### 2.5.4. Blood Sampling and Analysis

Blood samples were collected from an antecubital vein three times during both trials before exercise and immediately after completing the first and second bouts. The serum was obtained after 10 min of centrifugation (3000 rpm, 4 °C) and stored at −80 °C until further analysis. Blood glucose, lactate, serum insulin, myoglobin, total ketone body, and Beta-hydroxybutyric acid (BHB) concentrations were measured. Serum insulin, myoglobin, total ketone body, and BHB concentrations were measured using a clinical laboratory (SRL, Tokyo, Japan). Blood glucose and lactate concentrations were measured immediately after the blood was collected using a glucose analyzer (Freestyle, Nipro Inc., Osaka, Japan) and a lactate analyzer (Lactate Pro, Arkray, Inc., Kyoto, Japan).

#### 2.5.5. Perceived Fatigue

Participants were asked to rate their perceived fatigue using a visual analog scale (VAS) before exercise, immediately after completing the first and second bouts, 30 min after completing the exercise, and the morning after the exercise. The VAS consisted of a 10 cm line ranging from 0 to 10, where 0 indicated no fatigue at all and 10 indicated extreme fatigue [29].

#### 2.5.6. Statistical Analysis

Data are expressed as mean ± standard error. Time-course changes in the IGC, blood parameters, acetone levels in exhaled breath, and body composition were compared using two-way repeated-measures analysis of variance (ANOVA) to determine the interaction (trial × time) and main effects (trial and time). When ANOVA revealed a significant interaction or main effect, the Tukey–Kramer *post hoc* test was performed. A paired *t*-test was used to compare the nutritional intake variables and t-AUC values from the IGC between the HCHO and MCHO. A *p*-value < 0.05 was considered significant.

## 3. Results

### 3.1. IGC

**Figure 2:**
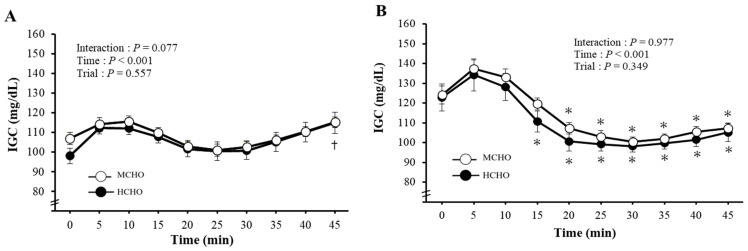

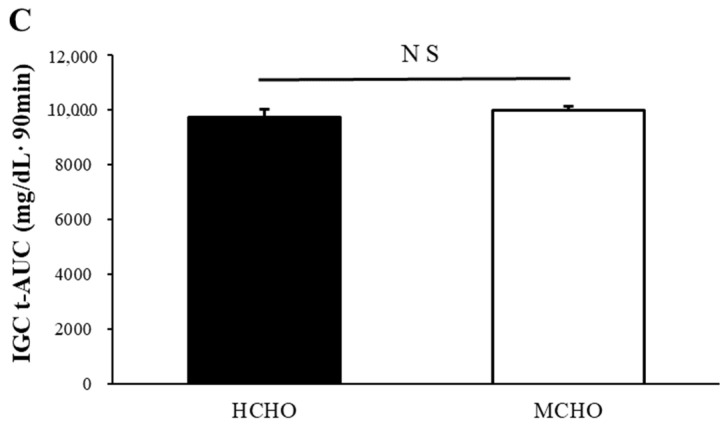
Changes in the IGC during the first bout (**A**), second bout (**B**), and the 2 × 45 min IGC t-AUC (**C**) during exercise. Values are mean ± SE. †: *p* < 0.05 vs. 0 min during the first bout. *: *p* < 0.05 vs. 5 min during the second bout. NS: No significant difference between trials.

Figure 2 shows the time-course changes in IGC every 5 min and t-AUC for two bouts × 45 min exercise. Two-way ANOVA indicated a significant (*p* < 0.05 unless state otherwise) main effect of time for the first bout and second bout (both *p* < 0.001). In the first bout, the IGC significantly increased after 45 min compared to 0 min in the HCHO. In the second bout, the IGC significantly decreased at 15 min in the HCHO and 20 min in the MCHO after reaching the peak level at 5 min. However, no significant differences were detected between the trials at any time point. The t-AUC value during exercise did not significantly differ between the HCHO (9719 ± 305 mg/dL·90 min) and MCHO (9991 ± 140 mg/dL·90 min, *p* = 0.370.

**Figure 3:**
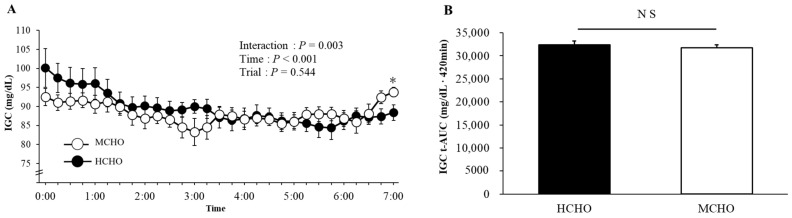
Changes in the IGC (**A**) and the t-AUC of IGC (**B**) during the night. Values are mean ± SE. *: *p* < 0.05 vs. 6:15. NS: No significant difference between trials.

Figure 3 shows the time-course changes in the average IGC (every 15 min) and t-AUC of the IGC for the nighttime (0:00–7:00) after exercise. A significant interaction (trial × time, *p* = 0.003) and a main effect of time (*p* < 0.001) were observed for the average IGC. Although the IGC significantly increased in the MCHO from 6:15 to 7:00, no significant differences between the trials were detected at any time point. The t-AUC value at night after exercise did not significantly differ between the HCHO (32,378 ± 873 mg/dL·420 min) and MCHO (31,749 ± 633 mg/dL·420 min, *p* = 0.456). In addition, the t-AUC value determined every 60 min the night after exercise did not significantly differ between the trials at any time point.

### 3.2. Blood Parameters

**Figure 4:**
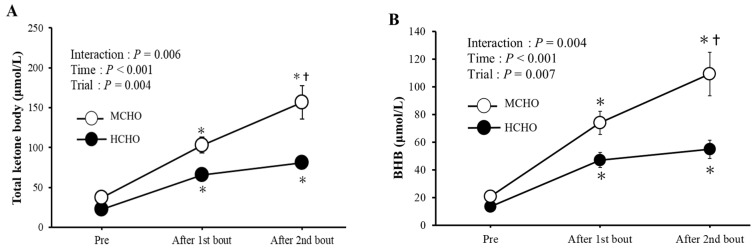
Changes in serum total ketone body (**A**) and Beta-hydroxybutyric acid (BHB) (**B**) concentrations. Values are mean ± SE. *: *p* < 0.05 vs. Pre. †: *p* < 0.05 vs. HCHO.

Figure 4 demonstrates the changes in serum total ketone body and BHB concentrations. A significant interaction and main effects of trial and time for serum total ketone body and BHB concentrations were found, and these values were significantly higher in MCHO than in HCHO after the second bout of exercise. 

Table 2 indicates the changes in blood glucose, serum insulin, lactate, and myoglobin concentrations. The baseline values (before exercise) of all parameters were similar between the trials. A significant main effect of time was observed for each parameter (all *p* < 0.01). Serum insulin concentrations significantly decreased immediately after the first and second bouts of exercise during both trials. However, these responses for all variables did not significantly differ between trials.

### 3.3. Acetone Levels in Exhaled Breath

**Figure 5:**
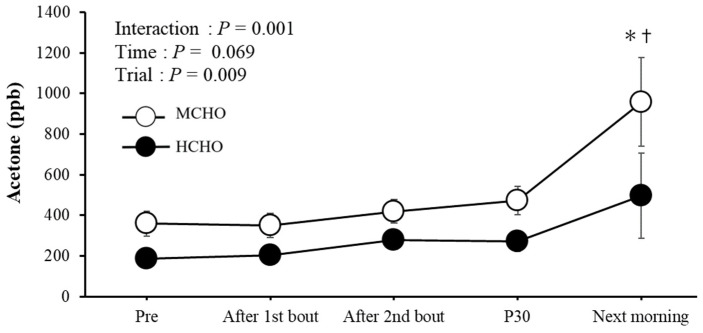
Changes in acetone levels in exhaled breath. Values are mean ± SE. *: *p* < 0.05 vs. Pre. †: *p* < 0.05 vs. HCHO.

Figure 5 shows the changes in acetone levels in exhaled breath. No significant differences between trials were observed at baseline. A significant interaction (trial × time, *p* = 0.001) and a main effect of trial (*p* = 0.009) were observed for acetone levels. The MCHO had higher levels than the HCHO in the morning after the exercise day. 

### 3.4. Perceived Fatigue

Baseline values of perceived fatigue did not significantly differ between trials. Two-way ANOVA demonstrated a significant main effect of trial (*p* = 0.015) and time (*p* < 0.001). The morning after the exercise, perceived fatigue was significantly lower compared to that after the second bout of exercise during both trials. However, it did not significantly differ between the trials at any time point. 

### 3.5. HR and RPE

Average HR during exercise was significantly higher during the second bout (HCHO: 159 ± 3 bpm, MCHO: 159 ± 3 bpm) than during the first bout of exercise (HCHO: 153 ± 4 bpm, MCHO: 151 ± 4 bpm) during both trials; however, no significant differences were observed between the trials in either bout. In addition, the RPE during exercise did not significantly differ between trials.

### 3.6. Body Composition

The changes in body composition are shown in Table 3. Baseline values did not significantly differ between the trials among any of the parameters. BW revealed a significant interaction (trial × time, *p* < 0.001) and time (*p* = 0.004). BW significantly decreased in the MCHO on day 4 compared to day 1. A significant interaction (trial × time) was detected for FFM (*p* < 0.001), and total body water (*p* = 0.006) and a significant main effect of time was detected for FFM (*p* = 0.003) and total body water (*p* < 0.001), respectively. FFM and total body water significantly decreased in the MCHO on days 3 and 4 compared to day 1. No significant interaction (trial × time, *p* = 0.369) or main effect of trial (*p* = 0.637) and time (*p* = 0.458) was detected for FM. 

## 4. Discussion

Our main finding is that IGC during and after running in a simulated soccer match did not significantly differ between the HCHO and MCHO. To the best of our knowledge, this was the first study to investigate continuous changes in glucose during the 24 h following a simulated soccer match in healthy active males who had consumed different quantities CHO.

### 4.1. IGC during Exercise 

Subjects in the MCHO consumed 4 g/kg BW/day CHO beginning 2 days before exercise, which was equal to the amount of CHO that soccer players usually consume on training days during the in-season period, including the day before a match [14]. In addition, the subjects in the MCHO had breakfast containing 1.4 g/kg BW CHO 90 min before starting the exercise. In the present study, no significant differences in blood lactate concentrations were observed immediately after the second bout of exercise between the trials. In addition, average HR during the first and second bouts of exercise were not significantly different between the trials. Thus, it appeared that physiological stress during exercise did not differ between the trials in the present study. Moreover, previous studies have reported that mean blood lactate concentrations during a soccer match are 2–10 mmol/L [7,30]. Therefore, the exercise intensity of the present study was comparable to an actual soccer match. 

The effect of low CHO intake on the glucose level of players during a soccer match is not consistent. O’Brien et al. [31] reported that 48 h of low CHO intake (3.5 g/kg BW/day) did not affect blood glucose concentrations during a Gaelic football match, which is similar to a soccer match. By contrast, Balsomet et al. [32] reported that post-exercise blood glucose concentrations were significantly lower in players 48 h after a low CHO diet (total energy intake: 2877 ± 654 kcal, CHO: 30% of total energy intake), which was estimated to be <3.5 g/kg BW CHO intake per day. Based on the present findings with no differences in IGC between the HCHO and MCHO trials, 4 g/kg BW/day CHO intake maintained the IGC during a simulated soccer match compared to 8 g/kg BW/day CHO intake. The reason for the lack of a difference in the IGC during exercise might be that the subjects consumed 1.4 g/kg BW CHO 90 min before exercise in the MCHO. The UEFA recommends consuming a CHO-rich meal (1–3 g/kg BW) 3–4 h before kick-off to ensure sufficient glycogen stores at the start of a soccer match [10]. Therefore, it is plausible that 1.4 g/kg BW CHO intake 90 min before exercise contributed to maintaining IGC during exercise in the MCHO.

Notably, IGC reached a peak 5 min after starting the second half during both trials. A 5–10 min delay exists between blood glucose concentration and IGC [33]. Therefore, IGC at 5 min during the second bout may reflect the blood glucose concentration at the onset of the second half of exercise. Once exercise is commenced, hepatic glucose production increases due to augmented glycogenolysis and gluconeogenesis [34,35]. In addition, if the exercise intensity is high, the blood glucose concentration is usually elevated as hepatic glucose output exceeds peripheral glucose uptake by muscles [36]. Thus, a transient elevation of IGC during the initial phase of the second half of exercise is understandable.

Exercise-induced fat metabolism is promoted under a lower stored glycogen condition to compensate for reduced glucose availability [37,38]. In the present study, this theory was supported, as serum total ketone body and BHB concentrations immediately after the second bout of exercise were higher in the MCHO than in the HCHO. Glucose homeostasis is tightly regulated [25,39]. Thus, it appears that serum ketone and BHB concentrations are more susceptible to a lower hepatic glycogen level than the IGC. 

### 4.2. IGC during the Night 

A unique aspect of the present study was that IGC was continuously monitored during the night (sleep) following the exercise. Consequently, IGC did not significantly differ during the night between the two trials. In addition, apparent hypoglycemia did not occur when the CHO intake was manipulated below the recommended level. However, one of eight subjects had an IGC level < 70 mg/dL at 5:45 (68 mg/dL) in the HCHO and at 3:00 (65 mg/dL) and 6:15 (69 mg/dL) in the MCHO, respectively. Thus, temporary hypoglycemia occurs during sleep after high-intensity exercise regardless of the CHO intake level. In previous studies, a high-CHO meal (CHO: 130 g) 45 min before bedtime increased non-rapid eye movement sleep and decreased light sleep and wakefulness compared to a low-CHO meal (CHO: 47 g) [40]. Grandner et al. [41] reported that lower CHO intake is associated with insomnia. In addition, hypoglycemia during sleep has an awakening effect [42]. Monitoring sympathetic and/or parasympathetic nervous system activity during sleep would be valuable for further study. 

The acetone levels in exhaled breath significantly increased in the MCHO the following morning. Acetone levels in exhaled breath are highly correlated with serum ketone body concentrations [43,44]. As fat mobilization is accelerated, acetone levels increase [45]. Therefore, the high levels in the MCHO reflected increased fat oxidation. In addition, a lower hepatic glycogen level may be involved. However, neither trial revealed a significant correlation between acetone levels the morning following exercise and the t-AUC of IGC during the night. 

### 4.3. Other Parameters and Study Limitations 

In the present study, serum myoglobin concentrations did not differ immediately after exercise between the two trials. An exercise-induced increase in the myoglobin concentration is an indirect marker of muscle damage [46,47]; however, detrimental effects of reduced CHO intake were not found in the present study. 

Our study had several limitations. First, we were unable to evaluate exercise performance. Second, the changes in hepatic levels of glycogen were not clear. Blood glucose concentrations and IGC reflect the hepatic level of glycogen rather than the muscle level [48]. However, to the best of our knowledge, no previous study has demonstrated the effect of different CHO intake levels on hepatic glycogen after a soccer match. Third, we utilized a simulated soccer match exercise on a treadmill. Therefore, the subjects did not replicate all of the movements during an actual soccer match. Finally, the sample size was limited due to the specific experimental design of individually manipulating CHO intake for consecutive days. Further investigations with a larger number of soccer players are required in the future.

## 5. Conclusions

Two days of consuming a different CHO intake level (8 or 4 g/kg BW/day CHO) did not significantly affect the IGC during a 90 min simulated soccer match. Moreover, the night IGC level post-exercise did not significantly differ between the two trials with different CHO intake levels. These findings indicate that glucose regulation during and after a simulated soccer match exercise is robust after consuming 4 g/kg BW/day CHO.

## Figures and Tables

**Table 1:** Total energy intake and macronutrients during the intervention period.

Variables	HCHO				MCHO			
	Day 1	Day 2	Day 3	Average	Day 1	Day 2	Day 3	Average
Energy (kcal)	2626 ± 118	2616 ± 128	2637 ± 114	2627 ± 120	2625 ± 119	2628 ± 123	2621 ± 121	2625 ± 120
CHO (g)	504.4 ± 22.1 *	503.7 ± 25.6 *	508.0 ± 22.2 *	505.3 ± 23.2 *	252.8 ± 12.0	254.8 ± 11.7	253.3 ± 11.3	253.8 ± 11.8
(g/kg)	7.9 ± 0.04 *	7.9 ± 0.06 *	8.0 ± 0.03 *	8.0 ± 0.04 *	4.0 ± 0.03	4.0 ± 0.04	4.0 ± 0.03	4.0 ± 0.04
(for energy)	76% *	77% *	77% *	77% *	39%	39%	39%	39%
Protein (g)	90.1 ± 4.2 *	88.9 ± 4.2 *	89.6 ± 3.7 *	89.7 ± 4.0 *	94.3 ± 4.2	95.5 ± 4.6	95.2 ± 4.3	95.0 ± 4.3
(g/kg)	1.4 ± 0.02	1.4 ± 0.02 *	1.4 ± 0.00 *	1.4 ± 0.01 *	1.5 ± 0.02	1.5 ± 0.01	1.5 ± 0.01	1.5 ± 0.00
(for energy)	14%	13% *	13%	13%	14%	14%	14%	14%
Fat (g)	28.2 ± 1.4 *	27.8 ± 1.1 *	28.1 ± 1.2 *	28.1 ± 1.2 *	137.9 ± 6.0	137.2 ± 6.4	136.5 ± 6.5	137.4 ± 6.2
(g/kg)	0.4 ± 0.00 *	0.4 ± 0.01 *	0.4 ± 0.01 *	0.4 ± 0.02 *	2.2 ± 0.02	2.1 ± 0.01	2.1 ± 0.01	2.2 ± 0.02
(for energy)	10% *	10% *	10% *	10% *	47%	47%	47%	47%

Values are means ± SE. CHO: Carbohydrate. HCHO: High carbohydrate diet. MCHO: Moderate carbohydrate diet. *: *p* < 0.05 vs. MCHO.

**Table 2:** Changes in blood parameters.

Variables	HCHO			MCHO		
	Pre	After 1st Bout	After 2nd Bout	Pre	After 2nd Bout	After 1st Bout
Blood glucose (mg/dL)	90 ± 4	105 ± 4 *	95 ± 4	86 ± 5	105 ± 5 *	96 ± 3
Insulin (µIU/mL)	20.83 ± 3.68	10.48 ± 2.39 *	4.21 ± 0.83 *	19.31 ± 4.06	9.29 ± 1.33 *	3.16 ± 0.42 *
Lactate (mmol/L)	2.2 ± 0.1	8.3 ± 1.0 *	6.4 ± 0.9 *	2.1 ± 0.2	7.2 ± 0.9 *	5.8 ± 0.9 *
Myoglobin (ng/mL)	34.0 ± 2.9	52.7 ± 5.1	120.0 ± 18.7 *	35.2 ± 3.8	54.2 ± 4.2	117.9 ± 12.7 *

Values are means ± SE. *: *p* < 0.05 vs. pre.

**Table 3:** Body composition during the intervention period.

		Day 1	Day 3	Day 4	Interaction (Trial × Time)	Main Effect	
						Trial	Time
Body weight (kg)	HCHO	63.5 ± 2.8	63.3 ± 3.0	63.2 ± 3.0	<0.001	0.410	0.004
	MCHO	63.9 ± 3.2	63.1 ± 3.2	62.7 ± 3.2 *			
Fat-free mass (kg)	HCHO	54.8 ± 2.3	54.4 ± 2.4	54.7 ± 2.7	<0.001	0.415	0.003
	MCHO	55.2 ± 2.5	54.0 ± 2.5 *	53.9 ± 2.5 *			
Fat mass (kg)	HCHO	8.7 ± 0.8	8.8 ± 0.9	8.8 ± 0.8	0.369	0.637	0.458
	MCHO	8.7 ± 0.9	9.1 ± 1.0	8.8 ± 0.9			
Total body water (L)	HCHO	40.0 ± 1.7	39.8 ± 1.7	39.8 ± 1.7	0.006	0.410	<0.001
	MCHO	40.3 ± 1.8	39.5 ± 1.8 *	39.4 ± 1.8 *			

Values are means ± SE. *: *p* < 0.05 vs. Day 1.

## Data Availability

Data are contained within the article.
